# Supplementary material for: Sex difference in the association between triglyceride and intracerebral bleeding risk after intravenous thrombolysis for acute ischemic stroke, a multi-center retrospective study
Source: PeerJ. 2024 Jun 24;12:e17558. doi: 10.7717/peerj.17558 (PMC11210480; doi:10.7717/peerj.17558)
Supplement: Supplemental Information 1 [file peerj-12-17558-s001.docx]

**Supplemental mateirals:**

Supplemental Table 1. Association of total cholesterol and the risk of sICH.

Supplemental Table 2. Association of LDL and the risk of sICH.

**Supplemental Table 1. Association of total cholesterol and the risk of sICH.**

|  | Unadjusted OR 95%CI | p-value | Age-NIHSS- adjusted OR 95%CI | p-value | Multivariable OR 95%CI | p-value |
| --- | --- | --- | --- | --- | --- | --- |
| **Men (n=628)** |  |  |  |  |  |  |
| Total cholesterol |  | 0.400 |  | 0.499 |  | 0.709 |
| Lowest tertile | Ref |  | Ref |  | Ref |  |
| Medium tertile | 0.75 (0.34 -1.63) |  | 0.80 (0.36-1.81) |  | 0.94(0.40-2.17) |  |
| Top tertile | 0.56 (0.24-1.32) |  | 0.58 (0.24-1.44) |  | 0.68(0.27-1.73) |  |
| Continuous (per mmol/l increase) | 0.74 (0.54-1.03) | 0.072 | 0.77 (0.55 -1.07) | 0.119 | 0.80(0.58-1.11) | 0.178 |
| **Women (n=329)** |  |  |  |  |  |  |
| Total cholesterol |  | 0.163 |  | 0.248 |  | 0.156 |
| Lowest tertile | Ref |  | Ref |  | Ref |  |
| Medium tertile | 0.22 (0.05 -1.05) |  | 0.26 (0.05 - 1.29) |  | 0.20 (0.04-1.016) |  |
| Top tertile | 0.74 (0.28-1.94) |  | 0.87 (0.32 - 2.36) |  | 0.86 (0.29-2.54) |  |
| Continuous (per mmol/l increase) | 0.93 (0.61-1.40) | 0.714 | 0.98 (0.66-1.46) | 0.921 | 0.97(0.64-1.47) | 0.883 |

**Note:** In men, adjusted for age, smoker, atrial fibrillation, NIHSS, stroke type, platelet count, and blood glucose (P<0.1 in the univariable analysis). In women, adjusted for age, hypertension, diabetes, atrial fibrillation, NIHSS, stroke type, and blood glucose (P<0.1 in the univariable analysis).

Abbreviations: CI: confidence interval; OR: odds ratio; LDL: low-density lipoprotein; NIHSS: National Institutes of Health Stroke Scale.

**Supplemental Table 2. Association of LDL and the risk of sICH.**

|  | Unadjusted  OR 95%CI | p-value | Age-NIHSS- adjusted OR 95%CI | p-value | Multivariable OR 95%CI | p-value |
| --- | --- | --- | --- | --- | --- | --- |
| **Men (n=628)** |  |  |  |  |  |  |
| LDL |  | 0.513 |  | 0.695 |  | 0.734 |
| Lowest tertile | Ref |  | Ref |  | Ref |  |
| Medium tertile | 0.92(0.43-1.97) |  | 0.95 (0.43-2.11) |  | 1.12(0.49-2.56) |  |
| Top tertile | 0.60 (0.25-0.45) |  | 0.68 (0.27-1.70) |  | 0.76(0.29-1.97) |  |
| Continuous (per mmol/l increase) | 0.74(0.52-1.07) | 0.106 | 0.80(0.56-1.14) | 0.220 | 0.83(0.59-1.17) | 0.285 |
| **Women (n=329)** |  |  |  |  |  |  |
| LDL |  | 0.420 |  | 0.607 |  | 0.698 |
| Lowest tertile | Ref |  | Ref |  | Ref |  |
| Medium tertile | 0.44(0.13-1.50) |  | 0.56 (0.16-1.98) |  | 0.56(0.14-2.16) |  |
| Top tertile | 1.78 (0.28-2.15) |  | 1.00 (0.35-2.89) |  | 0.80(0.25-2.55) |  |
| Continuous (per mmol/l increase) | 1.19 (0.83-1.71) | 0.344 | 1.23 (0.88-1.72) | 0.220 | 1.12(0.79-1.57) | 0.536 |

**Note:** In men, adjusted for age, smoker, atrial fibrillation, NIHSS, stroke type, platelet count, and blood glucose (P<0.1 in the univariable analysis). In women, adjusted for age, hypertension, diabetes, atrial fibrillation, NIHSS, stroke type, and blood glucose (P<0.1 in the univariable analysis).

Abbreviations: CI: confidence interval; OR: odds ratio; LDL: low-density lipoprotein; NIHSS: National Institutes of Health Stroke Scale.
